# MILAN Sky Survey, a dataset of raw deep sky images captured during one year with a Stellina automated telescope

**DOI:** 10.1016/j.dib.2023.109133

**Published:** 2023-04-11

**Authors:** Olivier Parisot, Patrik Hitzelberger, Pierrick Bruneau, Gilles Krebs, Christophe Destruel, Benoît Vandame

**Affiliations:** a Luxembourg Institute of Science and Technology, 5, avenue des Hauts-Fourneaux, L - 4362, Esch-sur-Alzette, Luxembourg; b Vaonis, 225 Rue Didier Daurat, 34170, Castelnau-le-Lez, France

**Keywords:** Electronically assisted astronomy, Deep Sky objects, Raw images

## Abstract

Modern automated telescopes allow to capture astronomical images in a reproducible way. During the MILAN research project (MachIne Learning for AstroNomy), we have observed deep sky with a Stellina observation station for twelve months from the Luxembourg Greater Region. Thus, we have captured raw images of more than 188 deep sky objects visible from the Northern Hemisphere (galaxies, stars clusters, nebulae, etc.), We have compiled and published this data as the MILAN Sky Survey dataset, allowing interested researchers, industry practitioners and citizens to reuse it.

**Specifications table**

**Table:** 

Subject	Astronomy and Astrophysics Space and Planetary Science
Specific subject area	Capture of deep sky objects images with an automated telescope for twelve months
Type of data	Images
How the data were acquired	Images were captured with a Stellina automated observation station from the Luxembourg Greater Region, with the observation sessions taking place over a total period of 12 months.
Data format	Raw images stored as 16-bit FITS files regrouped in ZIP archives (one per deep sky object capture session).
Description of data collection	The dataset compiles raw images acquired with a Stellina observation station for twelve months. More than 188 deep sky objects have been selected from existing catalogs: a representative set of deep sky objects from the Messier, NGC, IC, Sharpless, Barnard catalogs visible from the Northern Hemisphere during the period. Different types of objects were captured: emission & reflection nebulae, dark nebulae, planetary nebulae, galaxies, globular clusters, open clusters and even a comet. For each deep sky object, a zip file contains the raw images captured during the observation. Each raw image has an exposure time of 10 s. Raw images were not filtered.
Data source location	Institution: Luxembourg Institute of Science and Technology City/Town/Region: Esch-sur-Alzette Country: Luxembourg
Data accessibility	The large size of the dataset led us to divide it into several packages, one per month over one year. Repository name: MILAN project: raw images of deep sky objects captured with a Stellina observation station – [2022–03] Data identification number: zenodo.6865830 Direct URL to data: https://doi.org/10.5281/zenodo.6865830 Repository name: MILAN project: raw images of deep sky objects captured with a Stellina observation station – [2022–04] Data identification number: zenodo.6979484 Direct URL to data: https://doi.org/10.5281/zenodo.6979484 Repository name: MILAN project: raw images of deep sky objects captured with a Stellina observation station – [2022–05] Data identification number: zenodo.6874896 Direct URL to data: https://doi.org/10.5281/zenodo.6874896 Repository name: MILAN project: raw images of deep sky objects captured with a Stellina observation station – [2022–06] Data identification number: zenodo.6908651 Direct URL to data: https://doi.org/10.5281/zenodo.6908651 Repository name: MILAN project: raw images of deep sky objects captured with a Stellina observation station – [2022–07] Data identification number: zenodo.6976944 Direct URL to data: https://doi.org/10.5281/zenodo.6976944 Repository name: MILAN project: raw images of deep sky objects captured with a Stellina observation station – [2022–08] Data identification number: zenodo.7049839 Direct URL to data: https://doi.org/10.5281/zenodo.7049839 Repository name: MILAN project: raw images of deep sky objects captured with a Stellina observation station – [2022–09] Data identification number: zenodo.7115518 Direct URL to data: https://doi.org/10.5281/zenodo.7115518 Repository name: MILAN project: raw images of deep sky objects captured with a Stellina observation station – [2022–10] Data identification number: zenodo.7304607 Direct URL to data: https://doi.org/10.5281/zenodo.7304607 Repository name: MILAN project: raw images of deep sky objects captured with a Stellina observation station – [2022–11] Data identification number: zenodo.7399412 Direct URL to data: https://doi.org/10.5281/zenodo.7399412
	Repository name: MILAN project: raw images of deep sky objects captured with a Stellina observation station – [2022–12] Data identification number: zenodo.7498694 Direct URL to data: https://doi.org/10.5281/zenodo.7498694 Repository name: MILAN project: raw images of deep sky objects captured with a Stellina observation station – [2023–01] Data identification number: 10.5281/zenodo.7582149 Direct URL to data: https://doi.org/10.5281/zenodo.7582148 Repository name: MILAN project: raw images of deep sky objects captured with a Stellina observation station – [2023–02] Data identification number: 10.5281/zenodo.7625158 Direct URL to data: https://doi.org/10.5281/zenodo.7625158 Landing page, links to all datasets: https://publicationid.list.lu/milan-sky-survey-raw-images-captured-with-a-stellina-observation-station

## Value of the Data

• The dataset contains raw images of a large selection of deep sky objects (more than 188 deep sky objects visible from the Northern Hemisphere during a full year), heterogeneous (emission / reflection / dark / planetary nebulae, galaxies, globular clusters, open clusters) and representative of celestial targets that can be captured.
• Researchers, industrials, and citizens can use this data to apply and develop image post processing methods (e.g., frames filtering, demosaicing, alignment, stacking, color adjustments by photometry, histogram stretching, denoising).
• Data may be used by scientists for astrometry and photometry as complement of existing sky surveys obtained with professional ground telescopes.
• To our best knowledge, this dataset is the largest compilation of raw images captured by a portable automated observation station available to the public.

## 1 Objective

Nowadays, Electronically Assisted Astronomy (EAA) is widely applied by astronomers to observe deep sky objects (nebulae, galaxies, star clusters). By capturing images directly from a digital camera coupled to a telescope and applying soft image processing (raw images alignment and then stacking), this approach generates enhanced views of deep sky targets that can be displayed in near real-time on a screen (laptop, tablet, smartphone). EAA also allows to observe faint deep sky targets in adversarial outdoor conditions, for example in geographical areas heavily impacted by light pollution or during a night with the Moon. Faint celestial objects such as nebulae and galaxies are almost invisible by direct observation in an urban or suburban night sky; with EAA, they become impressive and detailed.

During the MILAN research project (MachIne Learning for AstroNomy), we have designed and tested innovative image processing techniques for EAA. Thus, an important step of the project was to collect images corresponding to what can be obtained with a portable equipment accessible to amateurs and under imperfect capture conditions (especially regarding light pollution [14]) – in a different way from what can be obtained with recent professional observatories (large diameters) located in ideal areas (e.g. mountains or desert).

For twelve months, we have captured images of 188 deep sky objects visible from the Northern Hemisphere (galaxies, stars clusters, nebulae, etc.) – and listed in well-known astronomical catalogs: Messier, New General Catalog (NGC), Index catalog (IC), Sharpless, Barnard [15].

Observations and captures were realized with a Stellina station (for more details, see Section 3), and the obtained images were compiled and published in a dataset.

## 2 Data Description

The dataset is composed of ZIP archives grouped and stored into several repositories – one per month (Table 1).

**Table 1:** List of ZIP archives per month. Each file is identified by the target code from well-known catalogs (Messier, NGC, IC, Sharpless, Barnard) and the observation date.

Month	Archive name (and count of raw images per archive)
3/2022 [1]	IC405-20220327.zip (390), IC443-20220324.zip (509), M100-20220325.zip (576), M105-20220325.zip (480), M44-20220324.zip (263), M46-20220323.zip (108), M51-20220310.zip (190), M64-20220323.zip (517), M67-20220308.zip (115), M81-20220308.zip (202), M82-20220308.zip (206), M86-20220308.zip (246), M94-20220325.zip (88), M95-20220327.zip (351), M96-20220309.zip (148), NGC1499-20220318.zip (257), NGC1579-20220327.zip (78), NGC2174-20220309.zip (473), NGC2244-20220323.zip (262), NGC2261-20220321.zip (367), NGC2371-20220323.zip (320), NGC2403-20220323.zip (263), NGC3628-20220324.zip (372), NGC4565-20220309.zip (360)

4/2022 [2]	M101-20220410.zip (245), M104-20220426.zip (243), M49-20220430.zip (802), M53-20220417.zip (356), M65-20220426.zip (325), M95-20220427.zip (350), NGC2371-20220416.zip (285), NGC2683-20220417.zip (400), NGC2903-20220416.zip (177), NGC4631-20220428.zip (306), NGC6543-20220416.zip (235)

5/2022 [3]	M12-20220513.zip (104), M13-20220517.zip (203), M5-20220509.zip (261), M61-20220502.zip (411), M82-20220509.zip (91), M92-20220531.zip (269), NGC3344-20220513.zip (486), NGC4535-20220508.zip (477), NGC4889-20220517.zip (517)

6/2022 [4]	M10-20220615.zip (197), M107-20220611.zip (115), M107-20220617.zip (129), M108-20220611.zip (93), M108-20220616.zip (119), M109-20220618.zip (261), M12-20220611.zip (116), M14-20220629.zip (134), M27-20220616.zip (262), M56-20220613.zip (114), M57-20220602.zip (103), M57-20220611.zip (79), M58-20220602.zip (317), M59-20220613.zip (503), M71-20220617.zip (138), M87-20220615.zip (202), NGC6633-20220615.zip (69), NGC6888-20220618.zip (378), NGC6946-20220620.zip (374), NGC7023-20220629.zip (282)

7/2022 [5]	IC1396-20220729.zip (427), IC4756-20220715.zip (73), IC5146-20220712.zip (336), M11-20220723.zip (131), M15-20220729.zip (140), M16-20220719.zip (121), M17-20220723.zip (194), M23-20220719.zip (110), M24-20220714.zip (56), M26-20220723.zip (116), M29-20220725.zip (119), M39-20220731.zip (94), M8-20220714.zip (122), NGC6823-20220725.zip (595), NGC6960-20220715.zip (385), NGC6992-20220716.zip (375), NGC7000-20220719.zip (639), NGC7789-20220719.zip (95)

8/2022 [6]	IC1795-20220809.zip (397), IC1805-20220828.zip (342), IC5070-20220810.zip (358), IC59-20220830.zip (352), M103-20220808.zip (151), M52-20220801.zip (206), M72-20220828.zip (76), M76-20220829.zip (192), NGC188-20220802.zip (70), NGC281-20220807.zip (357), NGC40-20220801.zip (185), NGC457-20220807.zip (328), NGC5907-20220809.zip (182), NGC6781-20220813.zip (219), NGC6826-20220807.zip (66), NGC6905-20220802.zip (183), NGC6979-20220829.zip (393), NGC7006-20220808.zip (285), NGC7008-20220803.zip (231), NGC7048-20220813.zip (195), NGC7129-20220808.zip (369), NGC7318-20220803.zip (340), NGC7635-20220802.zip (169), Sh2_101-20220801.zip (547)
9/2022 [7]	Barnard142_143-20220922.zip (265), IC342-20220919.zip (447), IC5070-20220922.zip (285), M110-20220904.zip (361), M2-20220904.zip (115), M31-20220901.zip (492), M33-20220919.zip (273), M74-20220921.zip (230), NGC6229-20220921.zip (250), NGC6883-20220911.zip (137), NGC7009-20220904.zip (160), NGC7331-20220911.zip (337), NGC7662-20220904.zip (184), NGC884_869-20220901.zip (108), Sh2_129-20220922.zip (230), Sh2_155-20220911.zip (200), Sh2_188-20220921.zip (259)

10/2022 [8]	Barnard133-20221009.zip (284), Barnard146-20221008.zip (172), IC10-20221006.zip (167), IC1318-20221009.zip (137), IC1318-20221026.zip (244), IC1848-20221006.zip (237), IC4955-20221008.zip (171), M34-20221008.zip (64), NGC1333-20221026.zip (231), NGC1342-20221009.zip (66), NGC185-20221026.zip (170), NGC246-20221011.zip (142), NGC246-20221024.zip (197), NGC559-20221006.zip (140), NGC663-20221006.zip (53), NGC6760-20221011.zip (238), NGC6891-20221008.zip (156), NGC6928-20221011.zip (135), NGC6934-20221009.zip (251), NGC7217-20221002.zip (552), NGC752-20221009.zip (59), NGC7606-20221011.zip (189), NGC7814-20221025.zip (371), NGC7822-20221026.zip (83), NGC891-20221002.zip (311), NGC925-20221008.zip (315)

11/2022 [9]	M45-20221113.zip (148), M77-20221101.zip (178), NGC1055-20221125.zip (298), NGC1342-20221125.zip (242), NGC7209-20221109.zip (66), NGC7293-20221113.zip (117), NGC7640-20221113.zip (401)

12/2022 [10]	IC2177-20221227.zip (203), IC348-20221217.zip (272), M1-20221216.zip (256), M35-20221212.zip (102), M37-20221216.zip (102), M42-20221216.zip (411), NGC1023-20221212.zip (217), NGC1245-20221210.zip (182), NGC1491-20221216.zip (252), NGC1579-20221212.zip (289), NGC2174-20221216.zip (362), NGC2419-20221217.zip (235), NGC488-20221217.zip (464), NGC6914-20221216.zip (321), NGC772-20221212.zip (271), NGC877-20221209.zip (128), NGC877-20221212.zip (216)

1/2023 [11]	IC410-20230118.zip (356), M38-20230113.zip (228), M46-20230130.zip (206), M50-20of230118.zip (108), M78-20230118.zip (132), NGC1027-20230130.zip (160), NGC1502-20230130.zip (177), NGC2022-20230130.zip (126), NGC2169-20230118.zip (89), NGC225-20230130.zip (211), NGC2360-20230130.zip (142), NGC864-20230111.zip (352)

2/2023 [12]	Barnard34-20230206.zip (281), IC417-20230212.zip (442), M48-20230207.zip (290), NGC1275-20230213.zip (320), NGC1788-20230206.zip (206), NGC1931-20230206.zip (412), NGC1961-20230208.zip (272), NGC2170-20230206.zip (222), NGC2264-20230206.zip (241), NGC2282-20230207.zip (109), NGC2420-20230208.zip (277), NGC2506-20230208.zip (286), NGC2841-20230212.zip (259), NGC3077-20230212.zip (250), NGC654-20230208.zip (252)

Each ZIP archive file contains a sequence of raw images for an observation session of a given celestial target. ZIP files are named with the respective target code and day of observation (format: NAME-YYYYMMDD.zip, for example: NGC1027-20230130.zip [11]).

As the same deep sky object can be identified with different identifiers coming from various catalogs, we only use one single identifier according to the following priority order: Messier, NGC, IC, Sharpless, Barnard [15]. Categories of deep sky objects are listed in (Table 2).

**Table 2:** List of deep sky objects per month. Each object is identified by its code in catalogs (Messier, NGC, IC, Sharpless, Barnard), and the type was extracted using the Stellarium software [18].

Period of observation	Deep sky objects and type
March 2022 [1]	IC405(Emission Nebula), IC443(Supernova Remanent), M100(Galaxy), M105(Galaxy), M3(Globular Cluster), M44(Open Cluster), M46(Open Cluster), M51(Galaxy), M64(Galaxy), M67(Open Cluster), M81(Galaxy), M82(Galaxy), M86(Galaxy), M94(Galaxy), M95(Galaxy), M96(Galaxy), NGC1499(HII Region), NGC1579(HII Region), NGC2174(HII Region), NGC2244(Open Cluster), NGC2261(Reflection Nebula), NGC2371(Planetary Nebula), NGC2403(Galaxy), NGC3628(Galaxy), NGC4565(Galaxy), NGC4565(Galaxy),

April 2022 [2]	M101(Galaxy), M104(Galaxy), M49(Galaxy), M53(Globular Cluster), M65(Galaxy), M95(Galaxy), NGC2371(Planetary Nebula), NGC2683(Galaxy), NGC2903(Galaxy), NGC4631(Galaxy), NGC6543(Planetary Nebula),

May 2022 [3]	M12(Globular Cluster), M13(Globular Cluster), M5(Globular Cluster), M61(Galaxy), M82(Galaxy), M92(Globular Cluster), NGC3344(Galaxy), NGC4535(Galaxy), NGC4889(Galaxy),

June 2022 [4]	M10(Globular Cluster), M107(Globular Cluster), M108(Galaxy), M109(Galaxy), M12(Globular Cluster), M14(Globular Cluster), M27(Planetary Nebula), M56(Globular Cluster), M57(Planetary Nebula), M58(Galaxy), M59(Galaxy), M71(Globular Cluster), M87(Galaxy), NGC6633(Open Cluster), NGC6888(Emission Nebula), NGC6946(Galaxy), NGC7023(Cluster associated with nebulosity)

July 2022 [5]	IC1396(Cluster associated with nebulosity), IC4756(Open Cluster), IC5146(Cluster associated with nebulosity), M11(Open Cluster), M15(Globular Cluster), M16(Cluster associated with nebulosity), M17(Cluster associated with nebulosity), M23(Open Cluster), M24(CL), M26(Open Cluster), M29(Open Cluster), M39(Open Cluster), M8(HII Region), NGC6823(Cluster associated with nebulosity), NGC6960(Supernova Remanent), NGC6992(Supernova Remanent), NGC7000(HII Region), NGC7789(Open Cluster)

August 2022 [6]	IC1795(HII Region), IC1805(Cluster associated with nebulosity), IC5070(HII Region), IC59(Reflection Nebula), M103(Open Cluster), M52(Open Cluster), M72(Globular Cluster), M76(Planetary Nebula), NGC188(Open Cluster), NGC281(HII Region), NGC40(Planetary Nebula), NGC457(Open Cluster), NGC6781(Planetary Nebula), NGC6826(Planetary Nebula), NGC6905(Planetary Nebula), NGC6979(Supernova Remanent), NGC7006(Globular Cluster), NGC7008(Planetary Nebula), NGC7048(Planetary Nebula), NGC7129(Cluster associated with nebulosity), NGC7318(IG), NGC7318(IG), NGC7318(IG), NGC7635(HII Region),

September 2022 [7]	Barnard142(Dark Nebula), IC342(Galaxy), IC5070(HII Region), M110(Galaxy), M2(Globular Cluster), M31(Galaxy), M33(Galaxy), M74(Galaxy), NGC6229(Globular Cluster), NGC6883(Open Cluster), NGC7009(Planetary Nebula), NGC7331(Galaxy), NGC7662(Planetary Nebula), NGC884(Open Cluster),

October 2022 [8]	Barnard133(Dark Nebula), Barnard146(Dark Nebula), IC10(Galaxy), IC1318(HII Region), IC1848(Open Cluster), IC4955(HII Region), M34(Open Cluster), NGC1333(Reflection Nebula), NGC1342(Open Cluster), NGC185(Galaxy), NGC246(Planetary Nebula), NGC559(Open Cluster), NGC663(Open Cluster), NGC6760(Globular Cluster), NGC6888(Emission Nebula), NGC6891(Planetary Nebula), NGC6928(IG), NGC6934(Globular Cluster), NGC7217(Galaxy), NGC752(Open Cluster), NGC7606(Galaxy), NGC7814(Galaxy), NGC7822(HII Region), NGC891(Galaxy), NGC925(Galaxy),

November 2022 [9]	M45(Cluster associated with nebulosity), M77(Galaxy), NGC1055(Galaxy), NGC1342(Open Cluster), NGC7209(Open Cluster), NGC7293(Planetary Nebula), NGC7640(Galaxy),

December 2022 [10]	IC2177(Reflection Nebula), IC348(Cluster associated with nebulosity), M1(Supernova Remanent), M35(Open Cluster), M37(Open Cluster), M42(HII Region), NGC1023(IG), NGC1245(Open Cluster), NGC1491(HII Region), NGC1579(HII Region), NGC2174(HII Region), NGC2419(Globular Cluster), NGC488(Galaxy), NGC6914(HII Region), NGC772(IG), NGC877(Galaxy),
January 2023 [11]	M38(Open Cluster), M46(Open Cluster), M50(Open Cluster), M78(Reflection Nebula), IC410(Cluster associated with nebulosity), NGC225(Open Cluster), NGC864(Galaxy), NGC1027(Open Cluster), NGC1502(Open Cluster), NGC2022(Planetary Nebula), NGC2169(Open Cluster), NGC2360(Open Cluster)

February 2023 [12]	Barnard34(Dark Nebula), IC417(HII Region), M48(Open Cluster), NGC654(Open Cluster), NGC1275(Galaxy), NGC1788(Reflection Nebula), NGC1931(Bipolar Nebula), NGC1961(Galaxy), NGC2170(Reflection Nebula), NGC2264(Open Cluster), NGC2282(Reflection Nebula), NGC2420(Open Cluster), NGC2506(Open Cluster), NGC2841(Galaxy), NGC3077(Galaxy)

In ZIP files, we have stored raw images in 16-bit FITS files (Flexible Image Transport System). This format is widely used in astronomy and files can be managed with any scientific image software like SIRIL [16] or with dedicated Python libraries like astropy [17].

Each FITS data file consists of a single Header Data Unit (HDU) with two elements: an ASCII text header and the binary data, i.e. the raw image as a single-channel matrix of 16-bits integers – obtaining a RGB image from this single-channel matrix can be done by demosaicing them (with the ‘RGGB’ pattern).

**Fig. 1:**
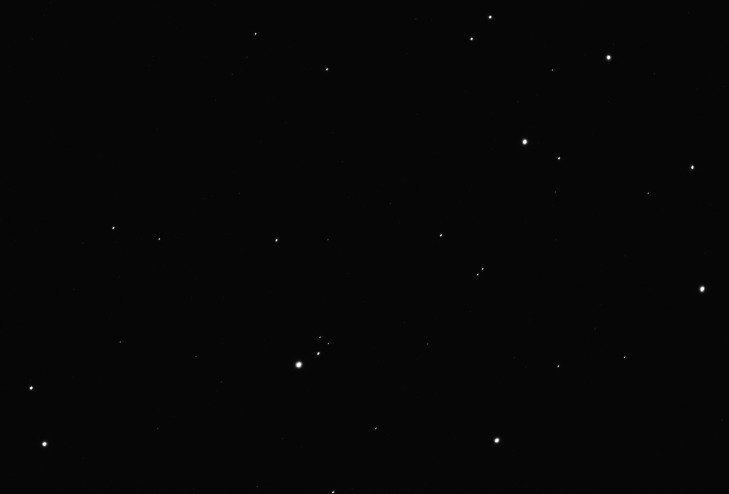
A raw image of M45 (Messier 45) displayed with the Gimp image software (M45-20221113.zip [11]). Colors, nebulosity, and faint stars are only visible after post-processing of the raw images (at least: demosaicing, alignment and stacking).

Raw images have a resolution of 3096 × 2080 pixels and correspond to a field of view of approximately 1° × 0.7° (we can see an example of raw image in Fig. 1).

## 3 Experimental Design, Hardware and Methods

### 3.1 Equipment

**Fig. 2:**
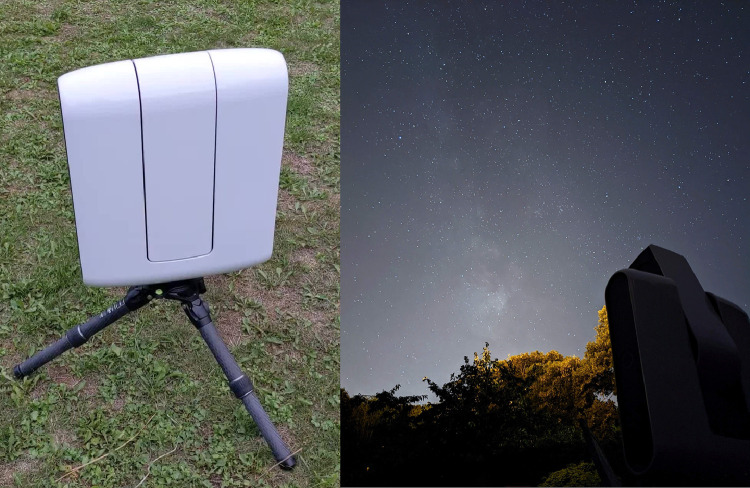
On the left, a photo of the Stellina station in off-mode during the day (20/9/2022). On the right, a photo of the Stellina station in action during the night (1/8/2022). These photos were captured with a Google 4a smartphone.

Raw images have been captured with a Stellina observation station, designed and commercialized by the VAONIS company (Fig. 2). This instrument is an improved and automated version of the well-known Short-tube 80 refractor, appreciated for its versatility by astronomers [13]. More precisely, Stellina is built with an apochromatic Extra-low Dispersion doublet with an aperture of 80 mm and a focal length of 400 mm - focal ratio of f/5. It is equipped with a Sony IMX 178 CMOS RGB sensor with a resolution of 6.4 million pixels (3096 × 2080 pixels). The Dawes Limit of the instrument is 1.45 arcseconds.

An anti-pollution filter (CLS type) is placed in front of the camera sensor. The observation station also has a fully automated alt-azimuth mount: setup, object tracking and focusing are also automated. Stellina also has an integrated field rotator that adapts to the target and allows the end-user to adjust the framing.

### 3.2 Deep sky targets selection

There are many tools to define a list of objects that can be captured.

One book has been very useful. In [19], the author details the different catalogs and the aspects to consider for making a relevant selection. For example, if we consider a famous catalog like NGC, which dates back to the 19th century, then we already have several thousands of objects which were visually accessible at a time when light pollution was much weaker than today. Nevertheless, it is important to choose targets that fit the characteristics of the optics and the sensor used, in our case objects that can be visualized using a Stellina station. We have therefore refined our selection based on [13], because it details the characteristics and the potential targets of the 80/400 refractor, an optical configuration very close to the Stellina. Moreover, before launching the observations, we simulated on the Stellarium software [18] the visualization of the pre-selected objects to check to their visibility and their position in the sky at the time of the observation.

**Fig. 3:**
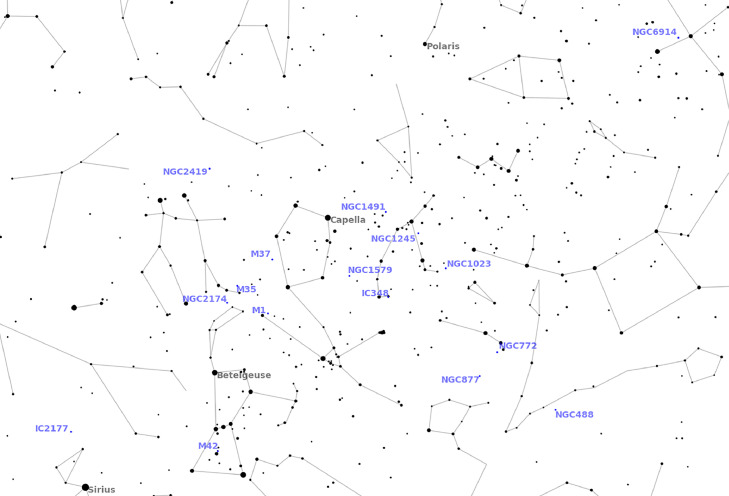
Projection on a celestial map of the deep sky objects targeted in December 2022. The map was realized with the skyfield package [20]. To assist in locating objects on the map, constellations lines are drawn, and names of well-known stars are shown.

As a result, we have selected various types of objects (Table 2, Fig. 3) – including known targets such as the Orion Nebula (Messier 42), but also little-known objects with low brightness reachable by an instrument like the Stellina observation station. In the end, the targeted object with the lowest magnitude is Messier 45 (1.2) and the targeted object with the highest magnitude is IC59 (13.33).

One last point to note: in practice, we got many more objects than those targeted. Indeed, the field of view of the instrument sometimes allowed to capture several objects at the same time. Let's take the case of the Messier 35 open cluster: it is located next to another cluster that was captured at the same time (NGC2158).

### 3.3 Procedure

**Fig. 4:**
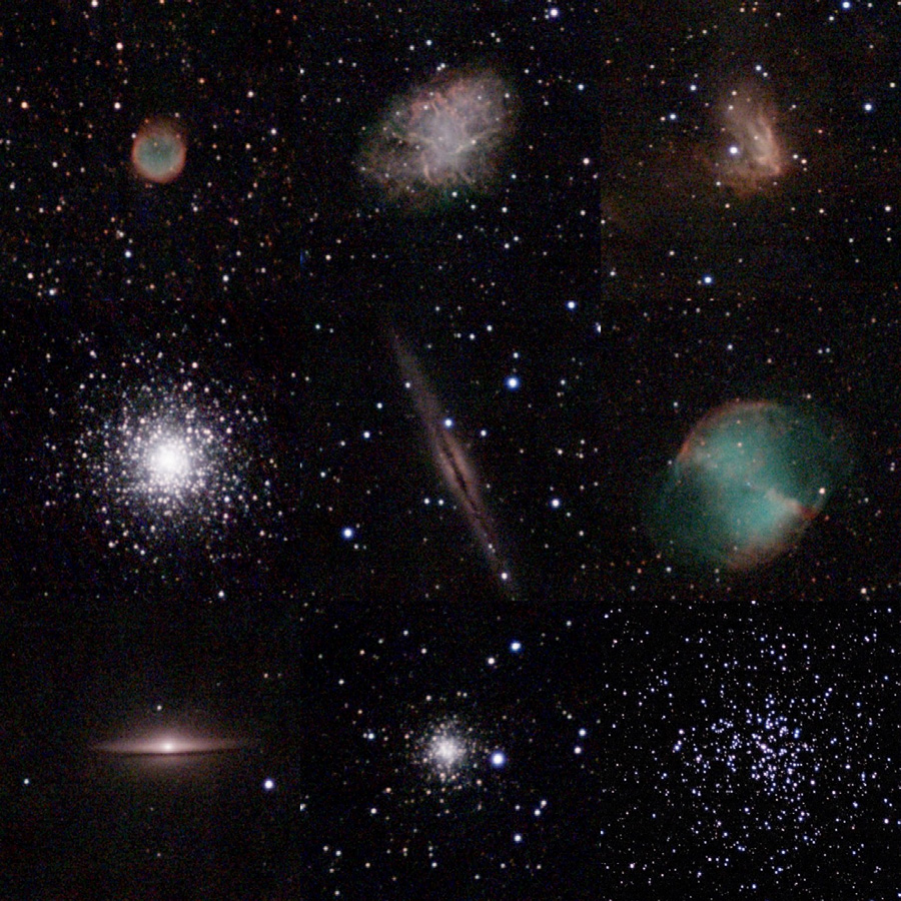
Samples of live post-processed RGB images produced by the Stellina telescope during the observations. From left to right and top to bottom: NGC6781, M1, NGC1491, M15, NGC891, M27, M4, NGC6934, M37. During the observations, these post-processed views allow to ensure that the capture process operates in good conditions.
- The default parameters of the Stellina observation station were applied: 10 s for exposition time and 20 dB for gain. These settings are optimized for tracking with an alt-azimuth mount [13].
- The total capture duration was selected according to the magnitude of the target. Approximately 100 raw images were captured for low magnitude stars clusters, while several hundreds of raw images were captured for high magnitude objects like nebulae.
- After capture sessions, raw images were copied from the Stellina telescope data storage and then compiled in zip files named with the target code and the observation date.

The following procedure was applied to capture images:

- Before capture sessions, the instrument was installed in a dark environment (no direct light) and properly balanced using a bubble level and held in the same position and location (longitude: 6.121, latitude: 49.142).
- During capture sessions, the sky was clear and allowed for reasonable quality acquisition. Authors were always near the instrument during observation, to avoid weather issues (wind, cloud, fog, rain) or disturbance (animals). The live-generated Stellina views allowed to control that the capture was going well (Fig. 4). If any issues appeared during the captures, then the data acquisition was stopped, and the data was not stored.

### 3.4 Challenges

Capturing data for twelve successive months was challenging. The weather is versatile in the observation site, and it was necessary to be available and ready as soon as the conditions were favorable. The duration of observation sessions is also highly variable from one season to another: shorter during the summer nights while the winter nights are much longer.

Dealing with these difficulties was the whole point of creating such a dataset, which corresponds to common observation conditions – contrary to the surveys produced from ideal locations.

## Ethics Statement

This work does not include any studies with human or animal subjects.

## CRediT authorship contribution statement

**Olivier Parisot:** Conceptualization, Methodology, Data curation, Supervision, Writing – original draft. **Patrik Hitzelberger:** Writing – review & editing. **Pierrick Bruneau:** Software, Writing – review & editing. **Gilles Krebs:** Writing – review & editing. **Christophe Destruel:** Writing – review & editing. **Benoît Vandame:** Writing – review & editing.

## Declaration of Competing Interest

The authors declare that they have no known competing financial interests or personal relationships that could have appeared to influence the work reported in this paper.

## Data Availability

MILAN Sky Survey: raw images captured with a Stellina observation station (Original data) (Zenodo).
